# Investigation of the origin of scatter components transmitted through anti-scatter grids in X-ray Digital Imaging system using Monte Carlo Simulation

**DOI:** 10.4314/ahs.v22i2.71

**Published:** 2022-06

**Authors:** Samson O Omondi, Peter K Msaki, Kazema R Ramadhan, Idrissa S Amour, Innocent J Lugendo

**Affiliations:** 1 Department of Radiography, College of Health Sciences, Jomo Kenyatta University of Agriculture and Technology, Nairobi, Kenya; 2 Department of Physics, University of Dar es Salaam, Dar es Salaam, Tanzania; 3 Department of Radiology and Imaging, Muhimbili University of Health and Allied Sciences, Dar es Salaam, Tanzania; 4 Department of Mathematics, University of Dar Es-Salaam, Dar es Salaam, Tanzania

**Keywords:** Monte Carlo simulation, Anti-scatter grids, image quality assessment, scatter correction

## Abstract

**Background:**

Projection diagnostic X-ray images are inherently affected by the masking effects of transmitted scatter. Spatially distributed transmitted scatter degrades image quality engendering need for effective scatter correction protocol.

**Objectives:**

To investigate origin of scatter components transmitted through anti-scatter grids to the detector of digital radiography system using Monte Carlo simulation.

**Methods:**

Over 107 photons were exposed through the reconstructed MC simulation phantom. Transmitted photons (primary and scatter) were scored as fluence, dose and deposited energy. Scatter components were investigated analytically over varying phantom thickness, tube kV and grid characteristics. Test disks were exposed as ROI embedded in phantom to evaluate the potential contrast improvement in image quality with the proposed technique.

**Results:**

Simulated and experimental results were comparable and in agreement with literature. SPR and SF mean values of 10.5, 0.314 and 7.96, 0.242 through grids of ratio 10:1 and 16:1 respectively was observed. Analysis of scatter components generation in object, grid's assembly, and fluorescent yields gave mean values of 0.815, 0.167 and 0.017, respectively. Image contrast was observed to increase with tube voltage and grid ratio.

**Conclusion:**

Achieving better image contrast, reduced patient dose and low scatter transmission while maintaining superior image quality, using grids with high grid ratio and selectivity is recommended.

## Introduction

Projection X-ray imaging has become indispensable for medical diagnosis notwithstanding the rapid development of non-ionizing diagnostic modalities such as Magnetic Resonance Imaging (MRI) and sonography. Of the several applications, chest X-ray (CXR) is the most indicated for diagnosing pulmonary diseases and viral pneumonia associated with Covid-19^1^. According to studies reported^2^, 44% of all X-ray diagnostic imaging procedures performed in 2006 worldwide were CXR and examination requests are on the increase. Digitization has led to replacement of film-based conventional CXR to digital radiography (DR) and CXR-Computed Tomography (CXR-CT) using flat panel detectors (FPDs). Quality of CXR images are degraded by several factors. Some of which include; detector system artifacts and low detective quantum efficiency (DQE)^3^,^4^, Rayleigh and Compton scatter photon fluence^5^–^7^, motion artifacts due to patient voluntary and involuntary motions^8^,^9^, amongst others. Scatter being the most degrading factor in diagnostic X-ray images, its effects which amongst others include, image blurring, decreased contrast and image artifacts as “cupping” effects needs correction^7^,^10^–^12^.

Pre-and post-acquisition scatter corrections methods such as anti-scatter grids (ASGs) and scatter kernels convolution respectively, have been demonstrated to improve image quality in projection isocentric CXR imaging^1^,^13^–^16^. However, the effectiveness of scatter subtraction by convolutions come at a cost of the high computational requirements which makes pre-acquisition methods more attractive for clinical applications. Despite its popularity, there is still a need to improve the performance of scatter suppression methods. However, in order to achieve enhanced performance there is need to investigate origin of the scatter components that consttute the scatter fraction transmitted to the detector and the distortion caused by the detector. This can be achieved using validated Monte Carlo simulations (MCs). Thus, the aim of this study is therefore to investigate the origin of scatter components transmitted through ASGs in digital X-ray imaging system geometry by MCs.

## Materials And Methods

### Monte Carlo simulation protocol

MCs of photon transport is based on random sampling process from the Klein-Nishina physical data that describe the interaction of photons in matter. Desired object geometry, density and composition are converted in simulation as voxel phantoms containing known materials and density composition or as reconstructed volumes and used as inputs. MCs code used had capability to assign appropriate energy for the simulation of polychromatic X-rays source spectra, pre-filtration, detector materials and thickness, beam collimation and ASGs. The simulation process conventionally follows these steps.

**Figure 1:**
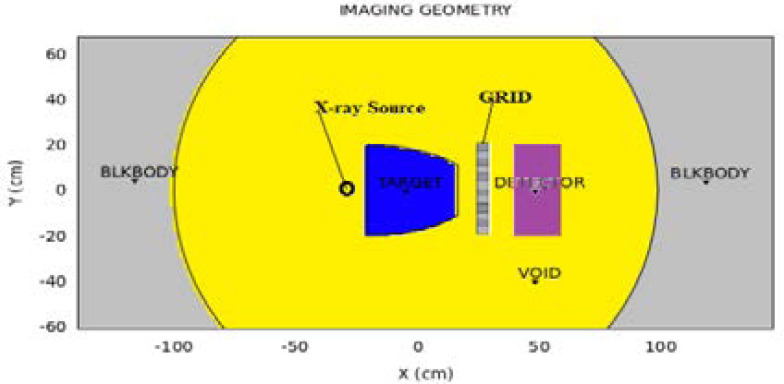
The simulation geometry set-up for photons transmission through ASGs during phantom irradiation.

First, conversion of the object into a MC phantom and allocation of CT numbers for each voxel which were used as input in image reconstructing algorithm which produced the projection data. Second, the simulation of the X-ray transport process was done using the inputs shown in the image geometry Figure 1. Two sets of photon fluence or deposited energy were obtaned. The first set obtained without the primary beam attenuator, transmitting photons in the detector registered as the total photon transmissions (*It*). Second set with the primary beam attenuator in place, detects all the photons deflected with an almost uniform angular distribution *φ*, for 0 < *φ* < *π*/2 recorded as the scatter transmitted estimate. (*Is*) The energy deposited in the detector was used for computation of the scatter distribution. And lastly, extraction of the primary estimate (*Ip*) was done through subtracting the scatter estimate (*Is*) from the raw projection data (*It*) in the spatial domain (*x,y*) using the expression:


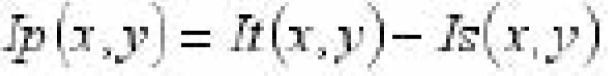



The energy of scattered photons was recorded as delayed low-intensity signals arriving at the detector. A second MCs was performed on the ASG to determine the transmissions of scatter from the originating from ASG structure. Produced and transmitted fluorescent X-rays, (*Ip*) and (*Is*) bombarding the detector was calculated using methods described 19,20.

### MCs Data acquisition

Using appropriate cards, MCs data were acquired and plots generated from the energy deposited, photon fluence and transmitted photons count. An estimate of scatter intensity was determined by simulating the physical path of transmitted and scattered photons through the object, ASGs into the image detector. The scan simulation time reduction techniques Russian Roulette (RR) and splitting were used to speed up the simulation process 20,21. Up to 107 photon histories were simulated and the statistical uncertainty calculated using Equation 2.



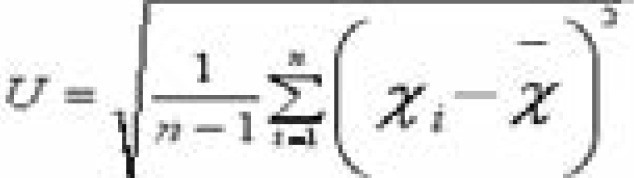



### Anti-scatter grids

Parallel ASGs were used in this study with the grid septa aligned to the central axis of X-ray source. To reduce the transmission of scatter photons while minimizing attenuation of the primary photons by the grid septa, appropriate alignment of ASGs was of utmost importance. Two grid performance parameters evaluated were SPR and SF using grid designs characterized by grid ratio (*r*) and frequency. (*N, lines / cm*)

### Image Contrast

Image quality was assessed by evaluating the contrast and contrast ratio of the diagnostic images. The effect of scatter correction on image quality, was investigated by exposing a tissue equivalent phantom with a copper test disk (∼ 5 cm diameter and 5 mm thick) embedded in the region of interest (ROI). The imaging was done on a DR C50 unit operating on automatic exposure control (AEC) at nominal voltage range of between 40–150 kV polychromatic X-ray beam. Image contrast was quantified in each image by measuring the signal difference due to the presence of the test disk. A circular ROI of approximately 6 cm in diameter was drawn within the borders of the disk signal and the image contrast calculated using Equation 3.


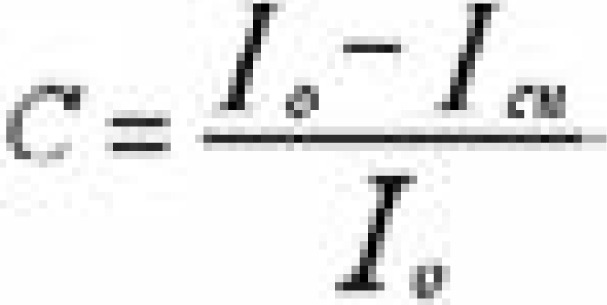


where, Io was the signal in the absence and Icu was signal in the presence of copper test disk.

### Scatter Fraction, Scatter to primary ratio determination

Phantom of varying thickness (10 to 50 cm) of cross-sectional area 30 × 30 cm2 was exposed with and without the anti-scatter grids. All measurements were acquired using a 110–140 kVp X-ray beam. The It without the beam stopper and Is with the stopper in place were obtained and used to calculate Ip according to Equation 1. From the values of It, Is and Ip the image quality parameters SF and SPR were calculated using relations described20. The source-to-image distance of the imaging system was maintained at 70 cm. These measurements were repeated for different grid features especially grid ratio between 10:1 to 16:1. The energy deposition wei/span>ghted by a quenching factor was scored based on the Birks' theory which relates the fluorescent yields, to the energy loss in the detector by Equation 4:


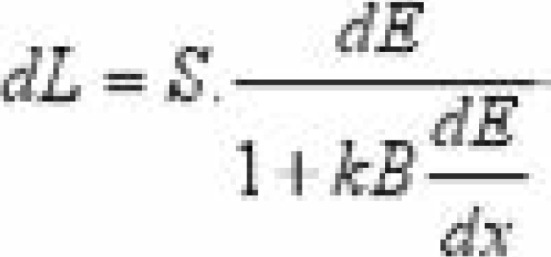


where *S* is the scintillation efficiency and *kB* is the Birks' constant. The total light yield in the detector is found by integrating equation 4, to obtain Equation 5;


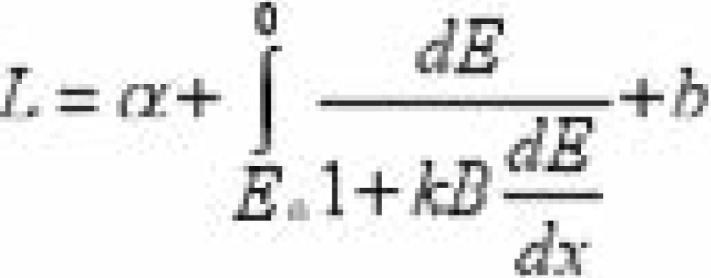


where, *α* and *b* are free parameters and *E*_0_ is the kinetic energy of photons.

## Results

### Anti-scatter grids performance

Table 1 show the investigated image quality parameters which define grid performance in scatter correction, SPR and SF values of four grids of varying septa frequency (*N*) and grid ratio (*r*)

**Table 1:** SPR and SF values for tissue equivalent phantom of varying thicknesses across four ASGs characteristics

Image Quality parameter	Grid	(Lines cm^-1^)	Ratio	Tissue equivalent Phantom Thickness (cm)				

				10	20	30	40	50
SF	*ASGN33R10*	33	10:1	0.23	0.27	0.33	0.36	0.38
SPR				2.6	6.6	13.1	14.4	15.8
SF	*ASGN40R10*	40	10:1	0.21	0.24	0.29	0.31	0.35
SPR				2.5	5.3	10.7	12.6	14.9
SF	*ASGN28R12*	28	12:1	0.20	0.23	0.27	0.29	0.32
SPR				2.2	4.8	9.8	11.4	13.6
SF	*ASGN36R16*	36	16:1	0.19	0.21	0.24	0.27	0.30
SPR				2.1	4.6	9.6	10.7	12.8

### Scatter components

**Figure 4:**
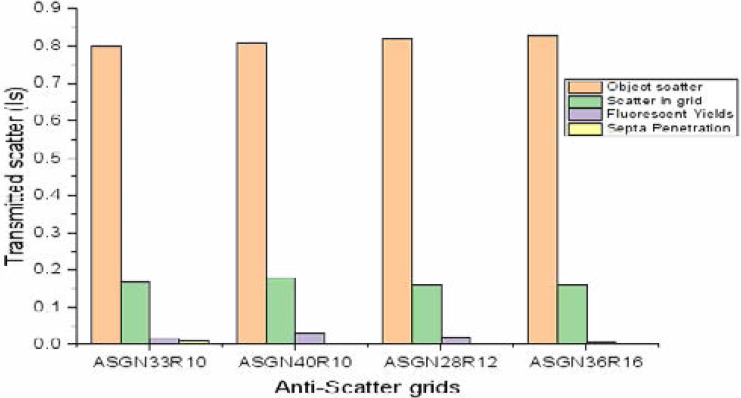
Components of transmitted scatter through selected four ASGs of varrying grid features (ratio and frequency).

Scatter occurrence is stochastic during radiation interaction with matter. Compton scattering dominates in biological tissues at tube voltages above 60 kVp. Analysis of scatter components showed scatter occurrence within the object, ASG assembly and as ploted in Figure 4.

### Contrast and contrast ratio assessment

**Figure 5:**
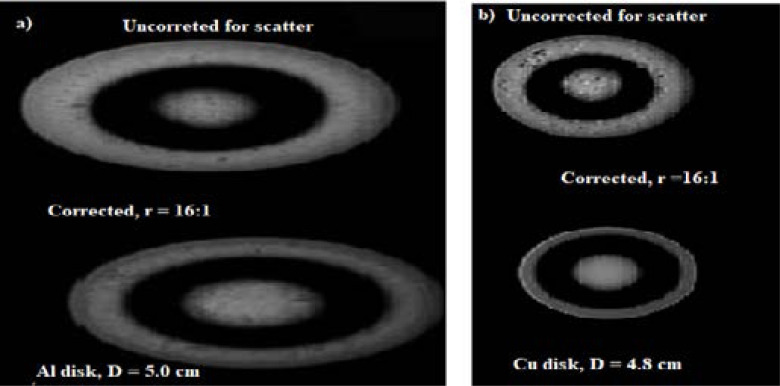
Results of uncorrected and corrected images for scatter of aluminum and copper disks exposed at 110 kV through grid ratio of 16:1

The images in Figure 5 show two test disks aluminum and copper exposed through ASG of ratio 16:1. Two sets of images are presented uncorrected and corrected for scatter.

## Discussion

**Figure 2:**
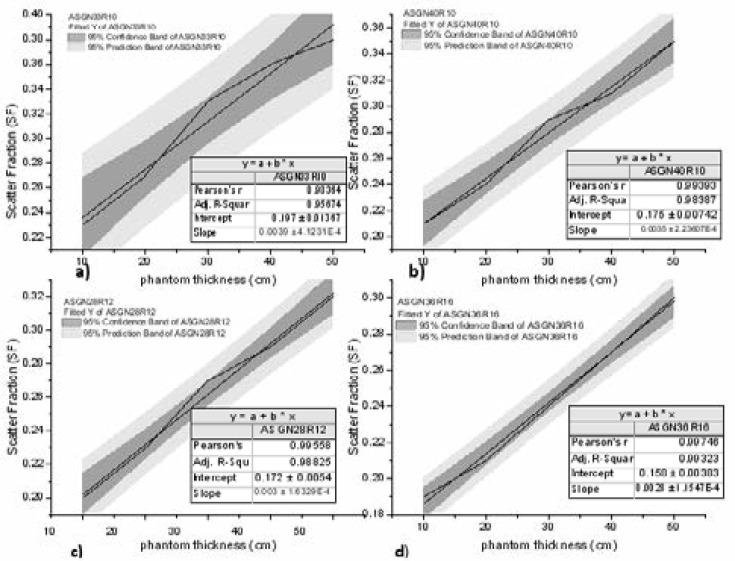
Scatter Fraction versus phantom thickness of four ASGs on a linear fit at 95% confidence band.

The results of this study presents a comprehensive investigation of the interplay between different ASGs designs and scatter correction capability in suppressing transmission of scatter photons in DR systems. At constant exposure energy (kV), scatter suppression increases with increasing grid ratio as expected because the angle at which the X-ray scatter impinges on the detector decreases with increasing grid ratio making high grid ratio grids more efficient for scatter rejection. Secondly the requirement for using short exposures with high grid ratio designs improves image contrast^23^. Additionally, use of grids enhances scatter reduction which depends on phantom thickness. The evaluation of the scatter correction by MCs through varying grid features has shown potential of significant reduction of scatter artifacts in projection X-ray imaging. SPR and SF were observed to increase with phantom thickness due to greater photon interactions as expected 6, 14. SPR has been shown to depend upon the examination conditions such as, body size and composition, field size, radiographic projection and system operating parameters, kV, atomic composition and thickness of detector and the position in the image plane^24^. The pearson correlation coefficient for higher grid ratio, Figure 2 (d) ASGN36R16 approaches unity at 0.987 compared to 0.981 of grid figure 2 (a) ASG33R10 at 95% confidence band.

SF range of 0.23 to 0.38 when phantom thickness increases from 10 to 50 cm, respectively was observed and this results from greater photon interaction probabilities within the phantom heterogeneous material. Depending on the phantom thickness, 4–8% more scattered radiation is transmitted through the grid ratio of 10:1 than for 16:1 since in the latter, more scatter is absorbed in the septa and the need for shorter exposures times for higher grid ratios therefore, improving image quality 23, 24. Simulation of photon transport through the phantom to the detector is stochastic and determined by the normal probability distribution function. The scoring of the energy deposited without grid and with grid conditions produced about 7–11% deviation in primary photons transmissions. Simulation of up-to 107 photon histories however, resulted into statistical uncertainty of less than 0.1%.

Scatter measurement took into account; the grid characteristics, number of scattered photons, photon histories and the deposited energy in the detector photo-peak window as well as the distribution of scatter which is source and object dependent, (*s*(*r, θ*)). The ASGN36R16 fitted at 95% confidence band produced a nearly linear increase of scatter fraction with the phantom thickness indicating need for scatter correction than attenuation correction for poly-energetic X-ray beam imaging. However, the main drawback of scatter suppression method in-spite of its effectiveness in scatter reduction is the primary photons cut-off by grid septa. Using higher grid ratio necessitates need for careful grid alignment to the X-ray beam iso-centre and use of shorter exposure times to minimize primary photon cut-off improving image contrast, Figure 5.

**Figure 3:**
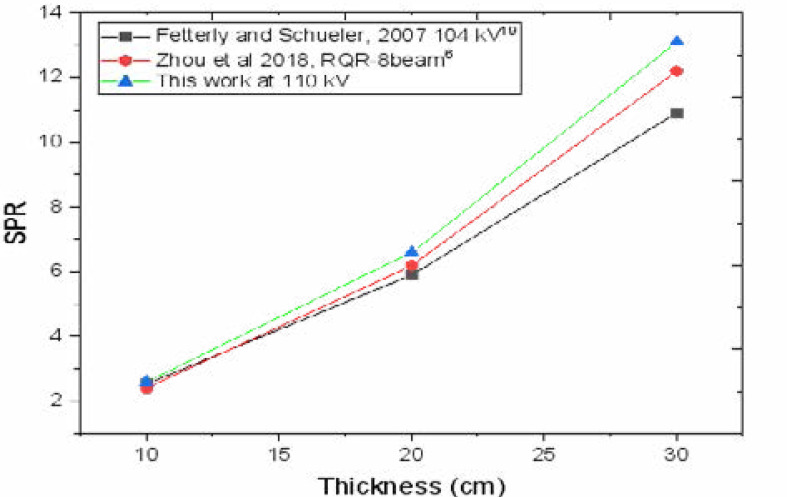
SPR as a function of phantom thickness exposed at 110 kV compared to two previous studies

The simulated and experimental results obtained were comparable and in agreement with literature with a Pearson correlation coefficient, r and adjusted R-square given as 0.987 and 0.974, respectively Figure 3. Scatter generation apart from the exposed object/phantom is observably significant within the grid assembly and from fluorescent yields constituting 16–18 % and 1–3%, respectively of total transmitted scatter. From Figure 4, use of lead in septa effectively attenuates scatter obliquely or tangentially incident on the grid completely eliminating septa penetration contribution to transmitted scatter which is consistent with previous studies^12^,^16^.

## Conclusion

Scatter generation is stochastic, its spatial distribution and transmitted components has been evaluated. To achieve a better image contrast, reduced patient dose, low scatter transmission while maintaining superior image quality, use of grids with high grid ratio and selectivity is recommended. The quantity of scatter absorbed by the grid septa increases with increasing grid ratio and phantom thickness as was expected. Scatter generation is significant in the object, grids assembly, from fluorescence and negligible from septa penetration. Transmitted scatter in object/phantom, anti-scatter grids assembly and fluorescent yields with mean values of 0.815, 0.167 and 0.017, respectively has been observed. The impact of this study would inform the development of improved grid designs with superior scatter suppression capabilities for improving quality diagnostic X-ray images.
